# Far‐red radiation stimulates dry mass partitioning to fruits by increasing fruit sink strength in tomato

**DOI:** 10.1111/nph.16805

**Published:** 2020-08-16

**Authors:** Yongran Ji, Diego Nuñez Ocaña, Daegeun Choe, Dorthe H. Larsen, Leo F. M. Marcelis, Ep Heuvelink

**Affiliations:** ^1^ Horticulture and Product Physiology Department of Plant Sciences Wageningen University & Research PO Box 16 Wageningen 6700AA the Netherlands

**Keywords:** dry mass partitioning, far red, LED lighting, sink strength, *Solanum lycopersicum* (tomato), sugar metabolism, sugar transportation

## Abstract

- Far‐red (FR) light promotes fruit growth by increasing dry mass partitioning to fruits, but the mechanism behind this is unknown. We hypothesise that it is due to an increased fruit sink strength as FR radiation enhances sugar transportation and metabolism.
- Tomato plants were grown with or without 50–80 μmol m^−2^ s^−1^ of FR radiation added to a common background 150–170 μmol m^−2^ s^−1^ red + blue light‐emitting diode lighting. Potential fruit growth, achieved by pruning each truss to one remaining fruit, was measured to quantify fruit sink strength. Model simulation was conducted to test whether the measured fruit sink strength quantitatively explained the FR effect on dry mass partitioning. Starch, sucrose, fructose and glucose content were measured. Expression levels of key genes involved in sugar transportation and metabolism were determined.
- FR radiation increased fruit sink strength by 38%, which, in model simulation, led to an increased dry mass partitioned to fruits that quantitatively agreed very well with measured partitioning. FR radiation increased fruit sugar concentration and upregulated the expression of genes associated with both sugar transportation and metabolism.
- This is the first study to demonstrate that FR radiation stimulates dry mass partitioning to fruits mainly by increasing fruit sink strength via simultaneous upregulation of sugar transportation and metabolism.

## Introduction

The recent development of light‐emitting diodes (LED) has stimulated research on light quality to achieve higher crop productivity with more health benefits but less energy consumption (Pattison *et al*., 2018). Far‐red (FR) radiation promotes plant growth and development (Li & Kubota, 2009; Park & Runkle, 2017; Zhen & Bugbee, 2020). In tomato, which is a crop of both economical and scientific importance, FR radiation significantly increased the fraction of dry mass partitioned to fruits and this increase was shown to be the main explanation of yield increase under additional FR radiation (Ji *et al*., 2019).

The fraction of dry mass partitioned to fruits is strongly influenced by vegetative sink strength and fruit sink strength, the latter being the product of the sink strength of individual fruits and total fruit number (Heuvelink, 1997). Sink strength, quantified as the growth rate of an organ under nonlimiting assimilate supply (potential growth), is the intrinsic capacity of an organ to attract assimilates (Marcelis, 1996). Unlike fruit sink strength, the vegetative sink strength cannot be easily measured experimentally, as leaves may become deformed when exposed to prolonged low sink‐source ratios (Ho *et al*., 1982; Nederhoff *et al*., 1992; Heuvelink, 1996). The sink strength of a fruit is directly linked to its capability to: (1) unload assimilates into the fruit; and (2) optimise the utilisation and metabolism of imported assimilates (Osorio *et al*., 2014). Photosynthetic assimilates (sucrose) unloading from the phloem to the sink is usually facilitated symplasmically by sugar transporters (Carpaneto *et al*., 2005; Chen *et al*., 2012). For example, sugar transporter activities positively correlate with sugar accumulation in pea (Lu *et al*., 2019), Arabidopsis (Gottwald *et al*., 2000), tobacco (Bürkle *et al*., 1998) and maize (Slewinski *et al*., 2009). Upon transportation into sinks, sucrose can be degraded into glucose, fructose, or other derivatives (Ruan, 2014). An enhanced hydrolysis of sucrose in sink organs may increase yield (Baroja‐Fernández *et al*., 2009) by increasing the gradient of sucrose concentration from source to sink (Ho, 1996; Koch, 2004; Fridman *et al*., 2004) and enhancing cell growth and sugar accumulation (Jin *et al*., 2009). In tomato, the inclusion of the more‐efficient wild allele of *Lycopersicum Invertase 5* (*LIN5*) increases soluble solids in fruits (Gur & Zamir, 2004; Fridman *et al*., 2004; Zanor *et al*., 2009). Silencing *INVINH1*, which encodes a putative inhibitor of *LIN5*, increases seed weight and fruit hexose level (Jin *et al*., 2009). Starch is also an important part of sugar metabolism. APGase, for example, is a key regulatory enzyme of starch biosynthesis and its activity positively correlates with fruit sugar content in tomato (Petreikov *et al*., 2006).

To our knowledge, there has been no report on the effect of FR radiation on the sink strength of fruits. However, there are some results hinting towards a positive FR effect on fruit sink strength. For example, FR radiation increases the size and dry mass of individual fruits in tomato (Fanwoua *et al*., 2019; Ji *et al*., 2019; Kalaitzoglou *et al*., 2019). Furthermore, key genes (e.g. *LIN*, *AGPaseL1*, *AGPaseS1*, *STS1*, *STS2*, *SBE1*), involved in regulating fruit sugar transportation and metabolism, are associated with phytochromes, which are photoreceptors sensing FR radiation (Fridman, 2003; Kocal *et al*., 2008; Bianchetti *et al*., 2018). Collectively, these results warrant further study into the application of FR radiation to lift sink strength of fruits, as was recently suggested, to have great potential in yield increase (Fernie *et al*., 2020).

In this research, we aimed to identify the mechanism by which FR radiation affects dry mass partitioning to fruits. We hypothesise that FR radiation affects partitioning by increasing sink strength of individual fruits. Furthermore, we hypothesise that FR radiation stimulates sugar transportation into the fruits as well as sugar metabolism in the fruits, resulting in a higher sink strength. Tomato plants were grown in a glasshouse with or without additional FR. Potential fruit growth, achieved by pruning each truss to one remaining fruit, was measured to quantify fruit sink strength. We used model simulation and concluded that the measured FR‐enhanced fruit sink strength could quantitatively explain the FR‐enhanced dry mass partitioning to fruits. In a climate chamber experiment, we observed that FR radiation increased fruit sugar concentration and upregulated expression of genes associated with both sugar transportation and metabolism. We conclude that FR radiation stimulates dry mass partitioning to fruits mainly by increasing fruit sink strength via simultaneous upregulation of sugar transportation and metabolism.

## Materials and Methods

### Plant material and growth conditions

To determine the effect of FR radiation on fruit sink strength an experiment was conducted in a glasshouse at Wageningen University (52°N, 6°E, Wageningen, the Netherlands). Tomato (*Solanum lycopersicum* L. cv Moneymaker) seeds were sown in potting soil and seedlings were transplanted 2 wk later into Rockwool blocks. After 4 wk, uniform young plants with 11 visible leaves were transplanted into another glasshouse and grown with the high wire system. This glasshouse was divided into eight compartments (5.0 m × 2.5 m) separated by white plastic film. Two double gutters were placed in each compartment, 12 plants (planting density 3.4 plants m^−2^) were placed on each double gutter, including two border plants placed at each end of a gutter. The day : night temperature was maintained at 19.2 ± 0.5 : 17.1 ± 0.5°C. Daily average CO_2_ partial pressure and relative humidity were 680 ± 80 μbar and 79 ± 5%, respectively. Plants were irrigated with nutrient solution (electrical conductivity 2.1 dS m^−1^, pH 5.5) containing 1.2 mM NH_4_^+^, 7.2 mM K^+^, 4.0 mM Ca^2+^, 1.8 mM Mg^2+^, 12.4 mM NO_3_^−^, 3.3 mM SO_4_^2−^, 1.0 mM PO_4_^2−^, 35 μM Fe^3+^, 8.0 μM Mn^2+^, 5.0 μM Zn^2+^, 20 μM B, 0.5 μM Cu^2+^, 0.5 μM MoO_4_^2−^. The EC and pH level of the nutrient solution in the drainage were monitored twice a week. In addition to the use of bumblebees, manual pollination with a Vibri Vario electronic bee (Royal Brinkman, ’s Gravenzande, the Netherlands) was applied three times a week. For all plants, side shoots were pruned when visible. In the 2^nd^ half of the experiment, four, five or six old leaves per plant were removed four times. The dry mass of the removed parts was recorded and included in the calculation of total dry mass and dry mass partitioning.

A climate chamber experiment was conducted to determine the sugar concentration and expression level of key genes in sugar transportation and metabolism. Two climate chambers were each divided into four compartments, separated by white plastic film and six uniform plants were placed in each compartment. The day : night temperature was maintained at 19.9 ± 0.6°C : 18.4 ± 0.4°C. Daily average CO_2_ partial pressure and relative humidity were 488 ± 31 μbar and 75 ± 6%, respectively. The same irrigation and plant management practices were applied as for the glasshouse experiment, except that only manual pollination was applied in the climate chamber experiment.

### Experimental treatments

**Fig. 1:**
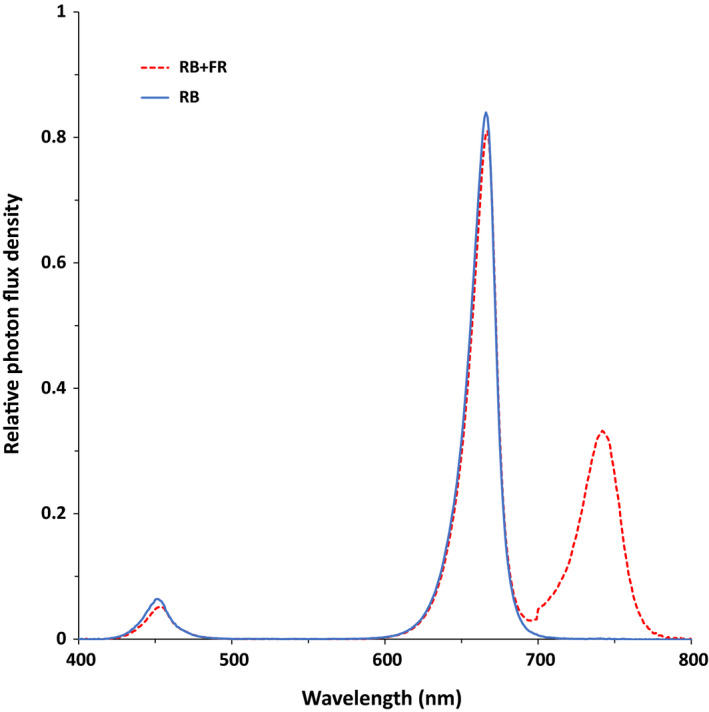
Spectral composition of red + blue (RB) and RB + far‐red (FR) light treatment provided by the LEDs measured at the top of the canopy.

There were two light treatments: red + blue (RB, R : B 95 : 5) without FR and RB + 80 μmol m^−2^ s^−1^ FR (RB + FR) (Fig. 1; Table 1). The spectral distribution and photon flux density (PFD) of the supplementary light was measured using a spectroradiometer (USB 2000 + UV‐VIS; Ocean Optics, Duiven, the Netherlands), on eight evenly distributed locations in each plot at the top of the canopy. Phytochrome photostationary state (PSS) in each treatment was calculated based on the measured spectra as the ratio of P_fr_ to the total of P_fr_ and P_r_ according to Sager *et al*. (1988). Photoperiod was 16 h. Hence, in the glasshouse experiment, the photoperiod was longer than the natural photoperiod, that is, LED lamps were switched on before sunrise and switched off after sunset. On average, solar daily photosynthetic photon flux density (PPFD, 400–700 nm) contributed *c. *18% to the total daily PPFD integral at canopy level in the glasshouse experiment (Supporting Information Fig. S1). All supplementary lighting was provided by overhead LED modules (RB: Greenpower TL‐DR/B‐150, FR: Greenpower PM‐FR‐150; Signify, Eindhoven, the Netherlands). The blue, red, and far‐red spectra peaked at 453, 666, and 735 nm, respectively. The height of the LED frames was adjusted weekly to maintain the desired PPFD at the top of the canopy (*c*. 170 μmol m^−2^ s^−1^ in the glasshouse experiment and *c*. 150 μmol m^−2^ s^−1^ in the climate chamber experiment; Table 1). When the LED frames reached the maximum height, the tops of the plants were lowered weekly (high wire cultivation system). A spectroradiometer was used to ensure that both PPFD and PSS values were maintained at the desired level every time the LED frame or the plant was adjusted.

**Table 1:** Photosynthetic photon flux density (PPFD), photon flux density (PFD) of far red, red: far red ratio and phytochrome photostationary state (PSS) value of the LED supplementary light measured at the top of canopy.

Experiment	Light treatment	PPFD (µmol m^−2^ s^−1^)	Far red (µmol m^−2^ s^−1^)	R : FR^1^	PSS
Glasshouse	RB	167 ± 5^2^	4 ± 0.5	42 ± 5.1	0.87
	RB + FR	170 ± 4	80 ± 4.2	2 ± 0.1	0.77
Climate chamber	RB	152 ± 1	2 ± 0.2	46 ± 5.2	0.88
	RB + FR	150 ± 3	51 ± 1.8	2 ± 0.1	0.78

^1^ For the calculations of ratios, PFD was integrated over 100 nm intervals for red (600–700 nm) and far red (700–800 nm).

^2^ All values are means ± SEM (*n* = 4). SEM of PSS was very small (<0.001) and therefore not shown.

In the glasshouse experiment, within each light treatment, plants were either not pruned or pruned to one, two, or five remaining fruits per truss (four plants per treatment in each of the four repetitions). Plants without fruit pruning were used to test the overall effect of FR radiation on partitioning to fruits, while those pruned to five fruits per truss were used to test whether partitioning was affected by FR radiation, independent of potential effects of FR radiation on fruit number. Plants with two fruits per truss were included to test whether one fruit per truss reflected potential growth. If there was no significant difference between fruit growth when fruit load was doubled (one fruit per truss vs two fruits per truss), then it can be assumed that observed fruit growth was potential fruit growth. The proximal fruit on each truss was removed at anthesis for all plants except those that received no fruit pruning. In the climate chamber experiment, there were five to six experimental plants in each of the four repetitions and all trusses were pruned at anthesis to one remaining fruit per truss to achieve potential fruit growth.

### Measurement of growth parameters

Leaf number (width > 1 cm) was determined weekly and numbers of flower buds, flowers (fully open flower) and fruits (visible set fruit with a diameter > 5 mm) on the 2^nd^, 3^rd^ and 4^th^ trusses were recorded three times a week for the unpruned plants and those with five fruits per truss. Fruit ripening was monitored three times a week with a hand‐held pigment analyser (PA1101; CP, Potsdam‐Golm, Germany). Two readings on the equatorial region of each tomato fruit were taken to estimate the normalised difference vegetation index (NDVI) and the normalised anthocyanin index (NAI). When a fruit reached a NDVI value lower than −0.65 and a NAI value higher than 0.4, it was harvested as fully ripe fruit (Farneti *et al*., 2013) and the date was recorded. At the end of the experiment, two plants with five fruits per truss and two plants with no pruning were destructively measured from each of the eight main plots to determine dry mass of fruits, stem, and leaves (dried in a ventilated oven for 24 h at 70°C and then 36 h at 105°C). For plants with two or five fruits per truss, two ripe fruits from 2^nd^, 3^rd^ and 4^th^ truss were selected randomly per plot to count the number of seeds per fruit, as seed number may also influence fruit growth. The dry mass of these fruits was also measured and added to the calculation of dry mass partitioning.

### Determination of potential fruit growth

Potential growth rate (g d^−1^) was calculated from nondestructive measurement of height (*h*) and diameter (*d*) of the remaining proximal fruits on the 2^nd^, 3^rd^, and 4^th^ truss of the plants with one or two fruits per truss (the 1^st^ fruit was removed at anthesis). Measurements were performed twice a week and the volume (*v*) of a tomato fruit was calculated with the following formula (Eqn 1) assuming the shape to be a spheroid (Li *et al*., 2015):

(Eqn 1) $$v=\frac{4}{3}\cdot \pi \cdot {\left(\frac{d}{2}\right)}^{2}\cdot \frac{h}{2}$$

Forty fruits varying in size were randomly collected from each light treatment to establish a light‐specific linear regression between fruit volume and fruit fresh mass (*R* ^2^ = 0.99 for both RB and RB + FR). In accordance with Wubs *et al*. (2012) a 4^th^‐degree polynomial function was established between fruit age and fruit dry matter content under each light treatment (*R* ^2^ = 0.78 for RB and *R* ^2^ = 0.90 for RB + FR) by randomly sampling two fruits every 3–4 d. These samples were collected from a separate set of plants grown alongside the experimental plants. With this function, fruit fresh mass was converted to fruit dry mass. The dry mass of each individual fruit was fitted as a function of fruit age (DAA) using a Gompertz function (Eqn 2):

(Eqn 2) $$W\left(t\right)=W_{\max}\cdot e^{‐e^{‐k(t‐t_{m})}}$$

where *W*(*t*) is the dry mass (g) of a fruit at age *t* (days after anthesis, DAA), *W*_max_ is the upper asymptote of fruit dry mass (g), *k* is the growth‐rate coefficient, and *t_m_* is the fruit age (DAA) at maximum growth rate. The derivative of this Gompertz function gives the fruit growth rate (FGR, g d^−1^) as a function of fruit age (*t*, DAA) (Eqn 3):

(Eqn 3) $$\text{FGR}\left(t\right)=W\left(t\right)\cdot W_{\max}\cdot k\cdot e^{‐k(t‐t_{m})}$$

Fitting of the growth curve was carried out for each individual fruit using a nonlinear mixed model, which considered that the measurements on one fruit are grouped, while assuming lower variation between measurements of one fruit than between different fruits (Li *et al*., 2015).

### Model simulation of sink strength and dry mass partitioning

A crop growth model was used to determine whether an observed effect of FR radiation on fruit sink strength could quantitatively explain observed effects on partitioning. First, the growth model was used to simulate the daily dry mass production of a whole tomato plant during the experimental period. Following the LINTUL approach, which describes dry mass accumulation as a function of light interception and light use efficiency (Haverkort *et al*., 2015), daily plant dry mass (GR_plant_, g d^−1^) production was calculated as (Eqn 4):

(Eqn 4) $${\text{GR}}_{\text{plant}}=0.33\cdot \left(1‐e^{\left(‐0.004\cdot {\text{CO}}_{2}\right)}\right)\cdot \left({\text{DPAR}}_{\text{tot}}\right)\cdot \left(1‐e^{(‐0.7\cdot \text{LAI)}}\right)$$

where CO_2_ is the daily average CO_2_ partial pressure (µbar) during photoperiod, DPAR_tot_ is total daily photosynthetically active radiation (PAR) integral on top of the canopy (mol m^−2^ d^−1^) and *LAI* is leaf area index. LAI was assumed to increase linearly from 0.7 to 3.4 in 30 d after transplantation (DAP) and was maintained at 3.4. The 0.7 and 3.4 values used here were based on the LAI measured at transplantation and the start of leaf pruning; 0.33 was a constant calculated from the calibration process to fit the reference treatment (RB light, five fruits per truss).

The daily fraction of dry mass partitioned to fruits (*F*_fruits_) was calculated as (Eqn 5):

(Eqn 5) $$F_{\text{fruits}}=\frac{{\text{ss}}_{\text{fruit}}}{{\text{ss}}_{\text{fruit}}+{\text{ss}}_{\text{veg}}}$$

where SS_fruit_ is fruit sink strength (g d^−1^) and SS_veg_ is vegetative sink strength (g d^−1^). SS_fruit_ was calculated as the sum of measured potential growth rate of every fruit present at a given day. As suggested by previous studies, a constant SS_veg_ can be assumed at constant daily average temperature for the simulation of *F*_fruits_ (Kano & van Gavel, 1988; Heuvelink, 1996). In this simulation, SS_veg_ was calibrated to be constant at a value of 3.4 g d^−1^ to fit the observed final fruit dry mass fraction in the reference treatment (RB light, five fruits per truss). According to a previous study, a SS_veg_ of 3.4 g d^−1^ was within the reasonable range for tomato plants (Heuvelink, 1996). A sensitivity analysis was conducted to test the effect of changes in SS_fruit_ and SS_veg_ on F_fruits_. SS_fruit_ and SS_veg_ values calibrated for the treatment with RB light pruned to five fruits per truss were used as the reference. Then we simulated the *F*_fruits_ when the SS_fruit_ was set to −50%, −30%, −10%, +10%, +30% and + 50% of the reference value, while maintaining the SS_veg_ reference value. Finally, we simulated *F*_fruits_ with the same changes made to SS_veg_, while maintaining SS_fruit_ at the reference value.

For simulation of plants for which no fruits were pruned, SS_fruit_ was increased by 1.5 times because the fruit number on plants with no pruning was, on average, 7.3–7.5 fruits per truss, which was 1.5 times that of plants pruned to five fruits per truss. The product of GR_plant_ and *F*_fruits_ gave the daily fruit dry mass production, which was then accumulated over the whole growth period to calculate total fruit dry mass. The ratio between the accumulated fruit dry mass and accumulated plant dry mass gave the simulated fraction of dry mass partitioned to fruits and was compared with the final fraction of dry mass partitioned to the fruits in plants with five fruits per truss or no pruning, grown under RB and RB + FR. With this approach, we ensured that the simulated fraction of dry mass partitioned to fruits at the end of the growth period was only a result of the differences in SS_fruit_. If this would result in differences between the simulated and observed *F*_fruits_ under a given treatment, these differences would then suggest that SS_veg_ was also affected.

### Carbohydrate analysis

In the climate chamber experiment, flowering time of each fruit was registered. One flower/fruit per plant (five or six plants per treatment repetition) was harvested at the stages of 0 (fully open flower), 10, 20, 30, 40, 50 or 60 DAA. Additionally, two fully ripen fruits, determined as described before, were harvested from each plant. At harvest, fruits were detached from the plant and were quickly sliced into two halves. One half was immediately frozen in liquid nitrogen for later use. The other half was weighed for fresh weight before being transferred to a ventilated oven for drying (24 h at 70°C and then 36 h at 105°C), after which the dry weight was measured.

Frozen tissue of each individual sample was ground mechanically into fine powder with liquid nitrogen. Then, equal weights of powdered tissues harvested at the same developmental stage from six replicate plants of the same compartment were pooled into one sample and mixed well. Glucose, fructose, sucrose and starch concentrations were measured as described by Plantenga *et al*. (2019) with an adaptation that 300 mg ground frozen fresh material from each pooled sample was weighed and mixed with 5 ml of 85% ethanol in a shaking water bath for 20 min at 80°C. After centrifugation at 8500 *g* for 5 min, 1 ml of the supernatant containing soluble sugars was vacuum dried using a Savant SpeedVac rotary evaporator (SPD2010; Thermo Fisher Scientific, Waltham, MA, USA) and then dissolved in 1 ml Milli‐Q water and diluted 50× for analysis of soluble sugars. Sucrose, fructose and glucose quantification was conducted using a high‐performance ion chromatograph (ICS‐5000; Thermo Fisher Scientific) with an anion CarboPac 2 × 250 mm exchange column (PA1; Thermo Fisher Scientific) at 25°C with 100 nM NaOH as eluent at the flow rate of 0.25 ml min^−1^. Pulsed amperometry was used for detection and Chromeleon (Thermo Fisher Scientific) was used for analysis of the chromatograms and quantification of sugar concentrations. The remaining pellet after sugar extraction was used for starch determination. After discarding the supernatant that contained the soluble sugars, the remaining pellet was washed three times with 80% ethanol, each time followed by 5 min centrifugation and removal of the supernatant. The remaining pellet was dried for 20 min in a SpeedVac rotary evaporator and resuspended in 2 ml 1 mg ml^−1^ thermostable α‐amylase solution (Serva Electrophoresis, Heidelberg, Germany) and incubated for 30 min at 90°C. Then, 1 ml of 0.5 mg ml^−1^ amyloglucosidase (10115; Sigma‐Aldrich) in 50 mM citrate buffer (pH 4.6) was added and the mixture incubated for 15 min at 60°C so that the starch in the sample was converted into glucose. After centrifugation for 5 min at 8500 ***g***, 1 ml of the supernatant was diluted 50× and was used for quantification of glucose content as described above.

### Gene expression analysis

Quantitative reverse transcription polymerase chain reaction (RT‐qPCR) was used to determine expression levels of target genes involved in sugar transportation (*SUTs*, *HTs*, *SWEETs*), sucrose synthase (*SUS*), invertase (*LINs*, *INVINH*), starch synthesis (*STSs*, *SBE*, *AgpL1*, *AgpS1*) and starch catabolism (*pGlcT1*, *pGlcT3*). Further information and primer sequences of the target genes are provided in Table S1. Fine powders of the pooled samples of the fresh frozen fruit material was used for RNA isolation using the CTAB method (Schultz *et al*., 1994). RNA quality was examined with 2% agarose gel. Furthermore, quality and concentration of the isolated RNA was tested with a spectrophotometer (DS‐11, DeNovix Wilmington, DE, USA). 200 ng of RNA was treated with RQ1 RNase‐free DNase kit (Promega, Madison, WI, USA) and synthesised to cDNA with the MultiScribe kit (Applied Biosystems, Foster City, CA, USA). The DNase treatment and cDNA synthesis were conducted according to the manufacturers’ manuals. The synthesised cDNA was diluted five times before use. The expression of target genes was analysed in a 10 μl system of 5 μl SYBR‐green master mix (Bio‐Rad, Hercules, CA, USA), 0.5 μl forward primer (10 μM), 0.5 μl reverse primer (10 μM), 3 μl Milli‐Q water and 1 μl cDNA using a thermal cycler (CFX96; Bio‐Rad) set at 95°C for 2 min followed by 40 cycles of 95°C for 5 s, 60°C for 20 s, 72°C for 15 s, and for a melt curve from 55°C to 95°C in a 0.5°C steps every 5 s. Absolute fluorescence data were analysed using LinRegPCR software (Ruijter *et al*., 2009) and was normalised against two references genes (*ACTIN* and *EFα1)*.

### Experimental set‐up and statistical analysis

For the glasshouse experiment, each light treatment was repeated four times, while the four pruning treatments were applied within the light treatments. Therefore, the experimental set‐up was a split‐plot design with light as the main factor and pruning as a subfactor. The climate chamber experiment was a randomised complete block design with each light treatment repeated four times. Growth parameters were analysed using Genstat (v.18; VSN International, London, UK). The assumptions of homogeneity and normality of the residuals were tested with Bartlett's test and Shapiro–Wilk test, respectively. Data satisfied the assumptions and were further analysed by analysis of variance (ANOVA). Parameters of the Gompertz growth function of each individual fruit were estimated with using the package saemix in R (R Core Team, 2013) and were subsequently analysed for differences between treatments with ANOVA in Genstat. Statistical differences between light treatments for sugar content and relative expression levels of target genes were tested at each sampling stage using Student’s *t*‐test in Genstat. All statistical tests were conducted at a probability level of *α* = 0.05.

## Results

### Far‐red radiation promotes dry mass partitioning to fruits

**Fig. 2:**
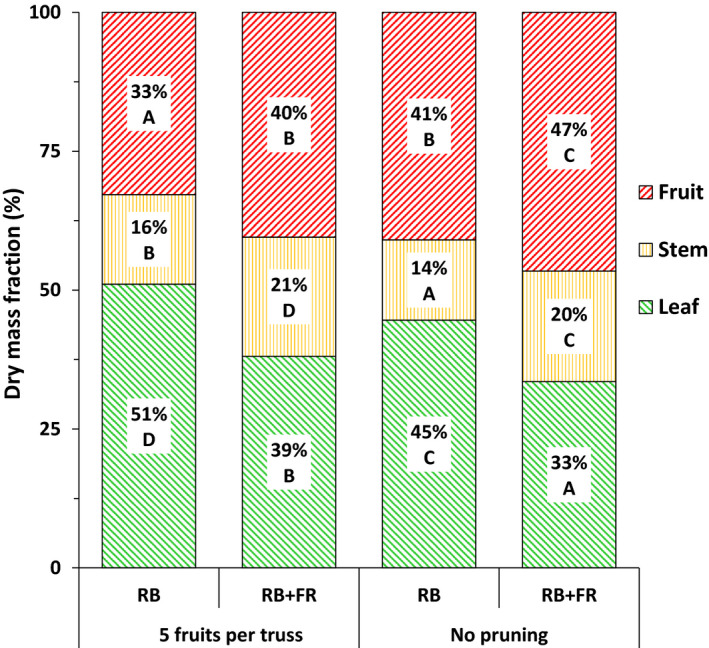
Effects of adding far‐red (FR) to red + blue (RB) light on the fraction of dry mass partitioned to fruits, stem and leaves in tomato (*Solanum lycopersicum*) plants where fruits were pruned to five fruits per truss or not pruned (7–8 fruits per truss). Data were based on cumulative dry mass of plants 90 d after transplanting. Different letters denote significant differences between treatments according to Fisher’s protected least significant difference (LSD) test conducted independently for fruit, stem, and leaf (*n* = 4, *α* = 0.05).

To study the effect of FR radiation on fruit number and dry mass partitioning to fruits, plants were either not pruned or pruned to five remaining fruits per truss. The plants without fruit pruning were used to test the overall effect of FR radiation on partitioning to fruits, while those pruned to five fruits per truss were used to test whether partitioning was affected by FR radiation, independent of the potential effects of FR radiation on fruit number. FR radiation significantly increased the fraction of dry mass partitioned to fruits and stems at the expense of that partitioned to leaves (Fig. 2). Also, FR radiation increased the dry mass of individual ripe fruits (Table 2). Pruning the trusses to five fruits per truss reduced the fraction of dry mass partitioned to fruits. Unpruned plants produced 42–43% more fruits and 20–30% more total fruit dry mass per plant, while total plant dry mass was not affected. FR radiation significantly accelerated fruit ripening, but had no effect on flowering time (Table 2). Total seed number per fruit, which may also influence fruit development, was not affected by either light or pruning treatment. Leaf appearance rate was not affected either (Fig. S2).

**Table 2:** Effects of adding far red (FR) to red + blue (RB) light and fruit pruning on total fruit number, dry mass of individual ripe fruits, total fruits (ripe and unripe fruits) and total plant dry mass, seed number of ripe fruits, flowering time of the 1^st^ truss (days after transplanting, DAP), fruit ripening time (days after anthesis, DAA) in tomato (*Solanum lycopersicum*).

Light	Fruit pruning	Total fruit number (per plant)^1^, ^2^	Dry mass of individual ripe fruits (g per fruit)	Total fruit dry mass (per plant)	Total plant dry mass (per plant)	Seed number (per fruit)	Flowering time (DAP)	Fruit ripening time (DAA)
RB	5 fruits per truss	62	2.7	92	277	154	22.2	71.6
	No pruning	88	2.2	123	302	155	22.6	71.7
RB + FR	5 fruits per truss	65	3.6	117	289	157	21.4	67.4
	No pruning	93	2.9	140	300	151	21.5	67.0
SEM (*n* = 4)		2.9	0.07	3.6	5.7	7.7	0.5	2.1
Light effect^3^		ns	**	**	ns	ns	ns	*
Pruning effect		*	**	*	ns	ns	ns	ns
Light × pruning		ns	ns	ns	ns	ns	ns	ns

^1^ The average number of fruits per truss ranged from 7.32 to 7.47 in the treatments without pruning, while the number of trusses per plant ranged between 12.0–12.4 for plants without pruning and 12.4–13.0 for those with five fruits per truss.

^2^ Total fruit number, dry mass of individual ripe fruits, total fruits (ripe and unripe fruits) and total plant dry mass measured 90 d after transplanting.

^3^ Asterisks denotes statistical significance tested with ANOVA: *, *P* < 0.05; **, *P* < 0.01; ns, not significant.

### Far‐red radiation increases sink strength of individual fruits

**Fig. 3:**
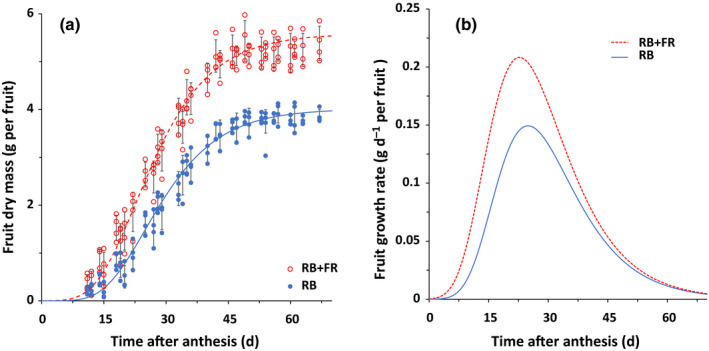
Effects of adding far‐red (FR) radiation to red + blue (RB) light on potential fruit growth (a) and potential fruit growth rate (b) in tomato (*Solanum lycopersicum*). Curves represent Gompertz function (a) and its derivative (b) fitted for RB + FR (dashed lines) and RB (solid lines) light conditions. Symbols represent measured fruit dry mass for RB + FR (open symbols) and RB (closed symbols) and error bar represents standard error of means (*n* = 4).

In order to quantify fruit sink strength, we pruned the tomato plants to one and two remaining fruits per truss to achieve a potential fruit growth condition in which fruit growth was not limited by the assimilate supply from the source leaves. Comparison between plants with one and two fruits per truss showed no difference in fruit growth, which means fruit growth was not affected even when fruit load was doubled (one fruit per truss vs two fruits per truss). Hence, we showed that fruit growth with one fruit per truss was potential fruit growth (Fig. S3). In the glasshouse experiment, FR radiation significantly promoted potential fruit growth (Fig. 3a). Potential fruit growth rate was higher under RB + FR throughout the whole growth period and reached a higher maximal growth rate at an earlier fruit age (Fig. 3b; Table S2). Similar FR radiation effect was also observed in the climate chamber experiment (Fig. S4).

### Increased sink strength of individual fruits explains the increase in fraction of dry mass partitioned to fruits

**Fig. 4:**
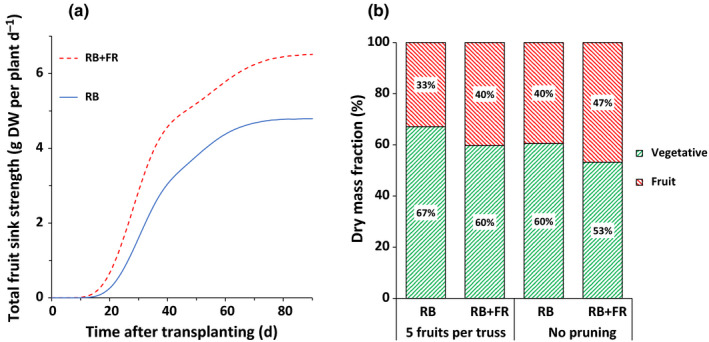
Simulated total fruit sink strength per plant for plants with five fruits per truss (a) and dry mass fraction of fruits and vegetative parts (b) of tomato (*Solanum lycopersicum*) plants grown with or without additional far‐red radiation. Curves represent fruit sink strength simulated for a fruit load of five fruits per truss. Both simulations were conducted for a period of 90 d after transplanting. RB, red + blue; FR, far red.

Fruit dry mass fraction was simulated with a model assuming proportionality between fruit dry mass fraction and the ratio between fruit sink strength and the sum of fruit and vegetative strength (Eqn 5). Using the measured data on flowering time and the potential growth rate of individual fruits, we calculated the total sink strength of all fruits together for plants with five fruits per truss grown with or without additional FR. The total fruit sink strength was increased by *c*. 38% by FR radiation and started to increase rapidly after flowering of the 1^st^ truss (*c*. 10 DAP) (Fig. 4a). The simulated fruit dry mass fraction was increased by *c*. 21% with additional FR radiation in both pruning scenarios (Fig. 4b). Simulation results agreed very well with the measured fraction of dry mass partitioned to fruits (Fig. 2). Using the scenario of plants with five fruits per truss grown with RB as reference, sensitivity analysis of the simulated dry mass partitioning to fruits showed that changes in both vegetative and fruit sink strength influenced dry mass partitioning to fruits (Table 3).

**Table 3:** Simulated fraction of dry mass partitioning to fruits in tomato (*Solanum lycopersicum*) when fruit sink strength (SS_fruit_) or vegetative sink strength (SS_veg_) was changed by −50, −30, −30, +10, +30 or +50%.

Parameter	Changes in input parameter relative to the control						
	−50%	−30%	−10%	Control	+10%	+30%	+50%
SS_fruit_	21%	26%	31%	33%	34%	38%	41%
SS_veg_	45%	39%	35%	33%	31%	28%	26%

The control refers to the simulated dry matter partitioning based on SS_fruit_ and SS_veg_ determined from the treatment with five fruits per plant under RB light.

### Far‐red radiation elevates fruit sugar content and sugar metabolism

**Fig. 5:**
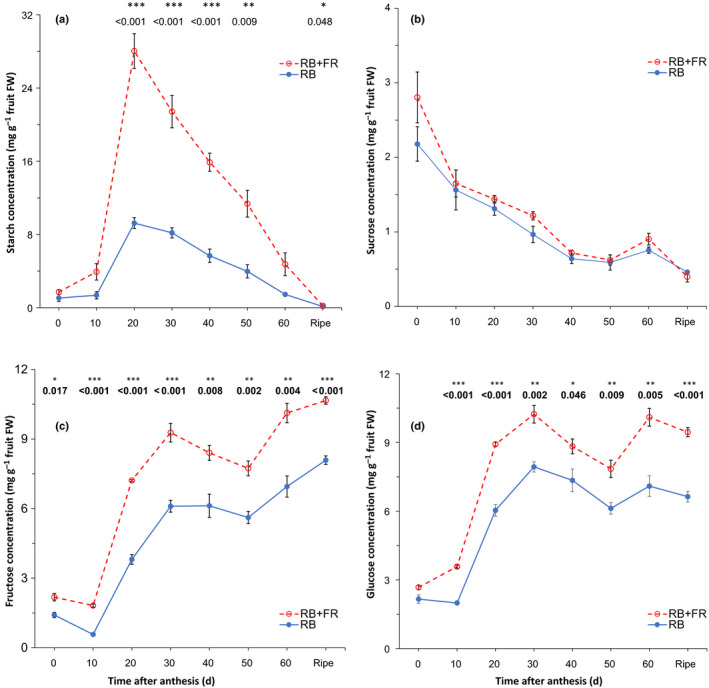
Effects of adding far‐red (FR) radiation to red + blue (RB) light on concentration of starch (a), sucrose (b), fructose (c) and glucose (d) in tomato (*Solanum lycopersicum*) fruits measured every 10 d after anthesis until fully ripe. Starch concentration is expressed as equivalent glucose concentration. Error bar represents standard error of means (*n* = 4). Asterisks denote statistically significant effects of FR radiation as tested with Student’s *t*‐test (*n* = 4; *, *P* < 0.05; **, *P* < 0.01; ***, *P* < 0.001).

To further understand the cause of the increase in fruit sink strength, we measured the concentrations of starch, sucrose, fructose and glucose throughout the growth of the fruits. Starch concentration increased rapidly until 20 DAA and then decreased almost linearly afterwards (Fig. 5a). Both rates of starch accumulation and break‐down were significantly accelerated by FR. Sucrose concentration gradually decreased during fruit development and no significant FR effect was observed (Fig. 5b). Both fructose and glucose concentrations increased during fruit growth and concentrations of both sugars were significantly higher in fruits grown with additional FR radiation after 10 DAA (Fig. 5c,d). Same FR effect was observed when concentrations were expressed on dry weight basis (Fig. S5).

### Far‐red radiation upregulates the expression of genes responsible for sugar transportation and metabolism in tomato fruit

**Fig. 6:**
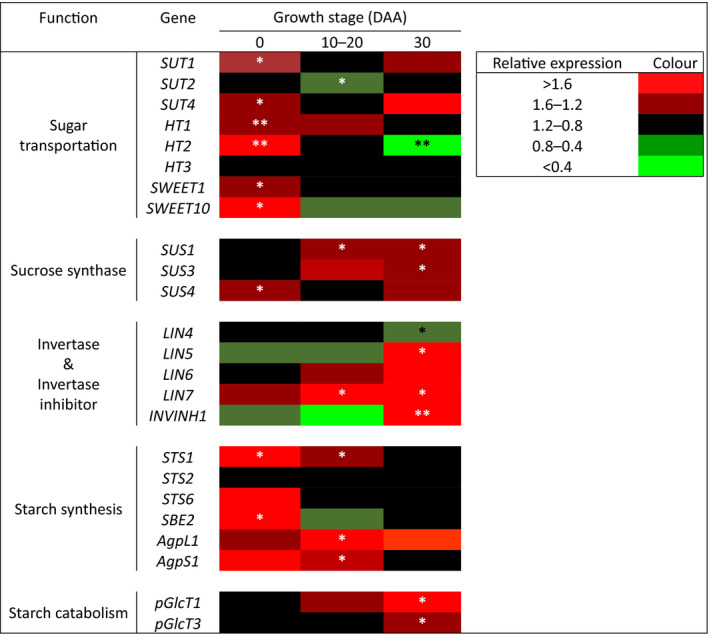
Effects of adding far‐red (FR) radiation to red + blue (RB) light on relative expression of genes related to sugar transportation, sucrose synthase, invertase, starch synthesis and starch catabolism in tomato (*Solanum lycopersicum*) flowers/fruits at 0, 10–20 and 30 d after anthesis (DAA). Different colours represent expression levels of each gene under RB + FR relative to that under RB. Asterisks denote statistically significant effects of FR radiation as tested with Student’s *t*‐test (*n* = 4; *, *P* < 0.05; **, *P* < 0.01).

To further explain the elevated sugar concentration, we measured the expression of key genes involved in sugar transportation and sugar metabolism at different growth stage of the fruits. At flowering stage (0 DAA), expression of genes encoding sucrose transporters, sucrose synthase and starch synthase were significantly upregulated by additional FR radiation (Fig. 6). During early fruit growth at 10–20 DAA, FR radiation significantly upregulated genes of sucrose synthase *SUS1*, invertase *LIN7* as well as ADP‐Glc pyrophosphorylase *AgpL1* and *AgpS1*. However, expression of sugar transporter *SUT2* was decreased by FR radiation. At 30 DAA, FR radiation significantly increased the expression of genes of sucrose synthases *SUS1 and SUS3*, the invertases *LIN5* and *LIN7* and starch catabolism *pGlcT1 and pGlcT3*. Interestingly, in addition to the increased expression of invertase genes, FR radiation also significantly increased the expression of *INVINH1*, which encodes a putative invertase inhibitor. In this developmental stage, FR radiation resulted in the downregulation of sugar transporter *HT2* and invertase *LIN4*.

## Discussion

### Far‐red radiation increases fruit sink strength and hence stimulates dry mass partitioning to fruits

FR radiation increased dry mass partitioning to fruits (Fig. 2) and total fruit dry mass per plant (Table 2), while PPFD was kept constant (Table 1). This finding agreed with recent reports on the effects of additional FR radiation on tomato growth and development (Ji *et al*., 2019; Kalaitzoglou *et al*., 2019; Zhang *et al*., 2019). Sink strength of individual fruits and total fruit number collectively determined total fruit sink strength and consequently influenced the fraction of dry mass partitioned to fruits (Marcelis, 1994; Heuvelink, 1997). FR radiation was reported to significantly increase size or mass of individual fruits (Ji *et al*., 2019; Kim *et al*., 2019). Indeed, we observed significantly higher individual fruit dry mass under additional FR radiation (Table 2). FR radiation significantly increased the potential growth rate and hence the sink strength of individual fruit (Fig. 3b). Model simulation, taking into account the observed increase in fruit sink strength, resulted in an increase in simulated dry mass partitioning to fruits (Fig. 4b). The sensitivity analysis of the model showed that both a higher fruit sink strength and a decreased vegetative sink strength can lead to increased dry mass partitioning to fruits (Table 3). Vegetative sink strength cannot be measured experimentally, hence it is difficult to evaluate directly whether it is affected by FR. However, in a separate experiment when 100 µmol m^−2^ s^−1^ FR radiation was added to 150 µmol m^−2^ s^−1^ RB LED lighting, soluble sugars in the leaves of 3‐wk‐old tomato plants increased from 7.9 mg g^−1^ DW to 19.1 mg g^−1^ DW. This FR‐increased soluble sugar was also reported by Courbier *et al*. (2020) and may suggest that FR radiation could affect vegetative sink strength. If that was true, we should have found significant differences between simulated and observed dry mass partitioning to fruits, as the simulation only took into account the observed increase in fruit sink strength while assuming a constant vegetative sink strength. However, the simulated results agreed very well with the observed result. Therefore, we reasoned that the FR radiation enhancement of fruit sink strength alone could explain the increase in fruit dry mass fraction.

Total plant dry mass, which reflected the source strength (dry matter production of the plant), was not influenced by FR radiation (Table 2). In a study with younger plants, an increase in source strength of the plants was observed and this increase was due to increased light interception (Kalaitzogolou *et al*., 2019). Here we started the light treatments when plants had already formed sufficient number of leaves and established an LAI of 3 within 4 wk. For a fruiting tomato canopy, an LAI of 3 usually means that 90% of the incident light will be intercepted (Heuvelink *et al*., 2018), leaving little room for FR radiation to affect total light interception (Ji *et al*., 2019). Total fruit number may also influence total fruit sink strength. FR radiation does not have a strong effect on total fruit number per plant (Ji *et al*., 2019; Kalaitzoglou *et al*., 2019; Kim *et al*., 2019; Table 2). Accelerated flowering is part of typical shade avoidance responses in many species (Ballaré & Pierik, 2017; Yuan *et al*., 2017) and may lead to a higher fruit number and consequently a larger total fruit sink size at a given time. We observed a trend of accelerated flowering of about 1–2 d caused by additional FR radiation, but this effect was not statistically significant (Table 2). Model simulation for plants grown under RB showed that advancing flowering time by 1 or even 2 d resulted in minor increase in the cumulative fraction of dry mass partitioned to fruits at the end of the experiment (Table S3). Hence, we argued that the FR effect on flowering time does not contribute significantly to the increase of dry mass partitioning to fruits in tomato.

Taken together, we concluded that the increase in fraction of assimilates partitioned into fruits under additional FR radiation is mainly due to an increase in fruit sink strength. To the best of our knowledge, this is the first study to demonstrate a strong FR effect on fruit sink strength and its contribution to dry mass partitioning.

### Far‐red radiation increases fruit sink strength by upregulating sugar transportation and metabolism in fruit

Sink strength is closely linked to the sugar metabolism in the sink organs (Osorio *et al*., 2014). Considering that the fruits in the present study were grown under nonlimiting assimilate supply, we reason that the sugar content in the fruits was mainly determined by the metabolic activities in the fruit. Tomato fruit typically switches from starch accumulation to hexose‐accumulation at around 15–20 d after anthesis (Ruan & Patrick, 1995). In agreement with this, starch concentration in the fruit kept increasing until 20 DAA (Fig. 5a). Starch may be considered as an overflow product when sugar concentration (especially sucrose) increases in the sink organ (Osorio *et al*., 2014). The substantial increase of starch concentration and upregulation of starch synthesis genes suggests a substantially increased amount of sugars are being transported into the fruit, and this was indeed supported by the upregulation of sugar transporters genes (Fig. 6). FR radiation significantly increased the fructose and glucose content in tomato fruits across the whole growth period, accompanied by an enhanced break‐down of starch (Fig. 5), which was also reported by Fanwoua *et al*. (2019). Indeed, after the fruit switched to hexose‐accumulation, we observed that FR radiation increased the expression of sucrose synthase and invertases in the fruits (Fig. 6), suggesting that FR radiation induced a stronger metabolism of the imported sucrose, thus allowing more sucrose to be imported into the fruit (Ho, 1996; Fridman *et al*., 2004; Koch, 2004). In accordance with our findings, specifically knocking‐down fruit‐localised phytochromes also (major FR‐sensing photoreceptors) upregulated *LIN5* and *LIN6*, which encode cell‐wall invertases crucial for sink activity in tomato fruits (Fridman, 2003; Kocal *et al*., 2008; Bianchetti *et al*., 2018). Interestingly, we also found that FR radiation increased the expression of *INVINH1*, which encodes an inhibitor of the above‐mentioned invertase Lin5 in tomato (Jin *et al*., 2009). Upregulation of *INVINH1* allows the tomato to regulate fruit development by capping cell‐wall invertase activity (Jin *et al*., 2009), thus further supporting that FR radiation elevated the invertase activities in tomato fruit. The sucrose concentration was not significantly affected by FR radiation (Fig. 5b). We observed a higher expression of sucrose transporters at flowering but not in later stages (Fig. 6). However, considering that FR radiation enhanced sugar metabolism and increased carbohydrate content, it is reasonable to argue that the import rate of sucrose should have been increased to maintain the same sucrose concentration in the fruit. This is further supported by the finding that *HY5*, a transcription factor known to directly enhance the expression of sucrose transporters *SWEET11* and *SWEET12* (Chen *et al*., 2016), was shown to be activated by FR radiation (van Gelderen *et al*., 2018). Interestingly, FR radiation also accelerated fruit ripening (Table 2). This is in agreement with studies in which knocking out *phyB1*, *phyAB1*, *phyB1B2* and *phyAB1B2* significantly reduced fruit ripening time by accelerating transition from mature green to breaker stage and that from breaker to red ripe stage (Gupta *et al*., 2014). Furthermore, phytochrome‐interacting factors (PIFs) have key regulatory roles in fruit ripening (Gramegna *et al*., 2019; Rosado *et al*., 2019). These results collectively suggested that phytochromes are important regulators of not only fruit growth but also fruit ripening.

### Conclusion

Additional FR radiation upregulated key genes involved in fruit sugar transportation and sugar metabolism, which resulted in a substantial elevation of fruit sink strength. When this FR effect on fruit sink strength was used in a model simulating dry mass partitioning, the model predicted very well the measured increase in dry matter partitioning to the fruits by FR radiation. Hence, we concluded that FR‐enhanced dry mass partitioning to fruits is primarily the result of an increased fruit sink strength.

## Author contributions

YJ, EH and LFMM conceptualised the research plan. YJ and DC designed and conducted the glasshouse experiment and analysed the data. YJ and DNO and DHL designed and conducted the climate chamber experiment and analysed the data. YJ wrote the manuscript. LFMM and EH initiated the project, acquired funding and guided the experimental design, data processing and interpretation and critically reviewed and edited the manuscript. All authors reviewed and approved the final manuscript.

## Supporting information


**Fig. S1** Solar daily light integral photosynthetically active radiation and its fraction of total photosynthetically active radiation in the glasshouse experiment.
**Fig. S2** Effects of additional far‐red radiation on leaf number in tomato (*Solanum lycopersicum*).
**Fig. S3** Comparison between tomato (*Solanum lycopersicum*) plants with one or two fruits per truss in the glasshouse experiment.
**Fig. S4** Effects of additional far‐red radiation on potential fruit growth in tomato (*Solanum lycopersicum*) in the climate chamber experiment.
**Fig. S5** Effects of additional far‐red radiation on fruit sugar content in tomato (*Solanum lycopersicum*) expressed on dry weight basis.
**Table S1** List of genes and corresponding forward and reverse primers used in the expression analysis.
**Table S2** Effects of additional far‐red radiation on the parameters of the Gompertz fruit growth curve of tomato (*Solanum lycopersicum*) in the glasshouse experiment.
**Table S3** Effect of earlier flowering on simulation of dry mass partitioning to fruits in tomato (*Solanum lycopersicum*) for a period of 90 d after transplanting.
Please note: Wiley Blackwell are not responsible for the content or functionality of any Supporting Information supplied by the authors. Any queries (other than missing material) should be directed to the *New Phytologist* Central Office.

## Data Availability

Source data supporting the findings of this study are available from the corresponding author upon request.
