# Supplementary material for: Far‐red radiation stimulates dry mass partitioning to fruits by increasing fruit sink strength in tomato
Source: New Phytol. 2020 Aug 16;228(6):1914–25. doi: 10.1111/nph.16805 (PMC7754386; doi:10.1111/nph.16805)
Supplement: Supplementary file 1 — Fig. S1 Solar daily light integral photosynthetically active radiation and its fraction of total photosynthetically active radiation in the glasshouse experiment. Fig. S2 Effects of additional far‐red radiation on leaf number in tomato (Solanum lycopersicum). Fig. S3 Comparison between tomato (Solanum lycopersicum) plants with one or two fruits per truss in the glasshouse experiment. Fig. S4 Effects of additional far‐red radiation on potential fruit growth in tomato (Solanum lycopersicum) in the climate chamber experiment. Fig. S5 Effects of additional far‐red radiation on fruit sugar content in tomato (Solanum lycopersicum) expressed on dry weight basis. Table S1 List of genes and corresponding forward and reverse primers used in the expression analysis. Table S2 Effects of additional far‐red radiation on the parameters of the Gompertz fruit growth curve of tomato (Solanum lycopersicum) in the glasshouse experiment. Table S3 Effect of earlier flowering on simulation of dry mass partitioning to fruits in tomato (Solanum lycopersicum) for a period of 90 d after transplanting. Please note: Wiley Blackwell are not responsible for the content or functionality of any Supporting Information supplied by the authors. Any queries (other than missing material) should be directed to the New Phytologist Central Office. [file NPH-228-1914-s001.pdf]

New Phytologist Supporting Information

Article title: Far-red radiation stimulates dry mass partitioning to fruits by increasing fruit sink strength in tomato

Authors: Yongran Ji, Diego Nuñez Ocaña, Daegeun Choe, Dorthe H. Larsen, Leo F. M. Marcelis, Ep Heuvelink

Article acceptance date: 3 July 2020.

The following Supporting Information is available for this article:

- Table S1 List of genes and corresponding forward and reverse primers used in the expression analysis
- Table S2 Effects of additional far red on the parameters of the Gompertz fruit growth curve of tomato (*Solanum lycopersicum*) in the greenhouse experiment.
- Table S3 Effect of earlier flowering on simulation of dry mass partitioning to fruits in tomato (*Solanum lycopersicum*) for a period of 90 days after transplanting.
- Fig.S1 Solar daily light integral photosynthetically active radiation and its fraction of total photosynthetically active radiation in the greenhouse experiment.
- Fig.S2 Effects of additional far red on leaf number in tomato (*Solanum lycopersicum*).
- Fig.S3 Comparison between tomato (*Solanum lycopersicum*) plants with one or two fruits per truss in the greenhouse experiment.
- Fig.S4 Effects of additional far red potential fruit growth in tomato (*Solanum lycopersicum*) in the climate chamber experiment.
- Fig.S5 Effects of additional far red on fruit sugar content in tomato (*Solanum lycopersicum*) expressed on dry weight basis.

Table S1 List of genes and corresponding forward and reverse primers used in the expression analysis

Locus	Gene	Forward primer	Reverse primer	Reference
Solyc01g109790	*AGPaseL1*	CGCTCACACAAGAGTTTCCA	CTAAGCGCGATCTTTCACCC	Bianchetti et al. 2018
Solyc07g056140	*AGPaseS1*	GCCCCAATCTACACCCAACC	CTGCCCCCATCAAAAGTGAG	Bianchetti et al. 2018
NM_001321306.1	*EFα1*	CGGCCACAGGGATTTCATCA	GGGTGGTAGCATCCATCTTGT	Expósito-Rodríguez et al. 2008
NM_001247106.2	*ACTIN*	ACTGGAATGGTGAAGGCTGG	CCCAGTTGCTGACAATACCG	Zhang et al. 2013
Solyc02g079220	*HT1 / STP1*	ATTGGAATTTCCGGGGGTGT	GGTGACAGTAGATGCCACCA	Shen, et al. 2019
Solyc09g075820	*HT2 / STP2*	CCTAGCTGGTTTAACGGCGA	GAACAGCCTGATTGGCGAAA	Shen, et al. 2019
Solyc07g006970	*HT3 / STP3*	ATTTGGCAACCAGGCAGTTC	TCATAACGAAAGCCGGTGCT	Shen, et al. 2019
Solyc03g083910	*LIN4 (TlVI)*	ACCATCCTACCCGATGGTCA	GTCCAAGCAGTAGTCGGGTC	Zhang et al. 2013
Solyc09g010080	*LIN5*	GGATGGGCTGGAATTCAAGGTA	AGCACTTCAACATCAGCCTGT	Zhang et al. 2013, Fridman & Zamir 2003, Liu et al. 2016
Solyc10g083290	*LIN6*	GCTCGAACCCGCTATCTACC	CGGGCTTGATCCACTTACGA	Zhang et al. 2013, Fridman & Zamir 2003, Liu et al. 2016
Solyc09g010090	*LIN7*	ATAATGCGAAGGGATGGGCT	CATCAGCCTGTGCTGGTGTG	Zhang et al. 2013, Fridman & Zamir 2003, Liu et al. 2016
Solyc03g112180	*INVINH1*	ACAGCAAGTATGCCAGAAGCA	ACAATGGCTCTACCAACATCAGA	Jin et al. 2009, Zhang et al. 2013, Shen et al. 2019
Solyc02g086160.2	*pGlcT1*	GCGGTCACTGGAGGAGATAG	TTCATGAAGCCGGTGTGTAA	Reuscher et al. 2014
Solyc07g020790.2	*pGlcT3*	GGTTGGATTGCTGATGGAGT	GCAGGAGAAACCTCTGCAAC	Reuscher et al. 2014
Solyc04g082400	*SBE2*	GTAAGCCAGCCATTCCACAC	TGGAAGGGCGAGGGTATTTG	Bianchetti et al. 2018
Solyc08g083320	*STS1*	TGAATGCGATGTTGTTGACCC	GCTCCTAAGCCCAATAGCAAC	Bianchetti et al. 2018
Solyc03g083090	*STS2*	CTGTTGACCTTGATGTGCGGG	GAGAAAGCAAAGTGAAGCGAAAC	Bianchetti et al. 2018
Solyc02g088000	*STS6*	ATGTGGTGGGGTCTGCTATG	AAAGGACCACGACCCTGATG	Bianchetti et al. 2018
Solyc12g040700	*SUS1*	CTTGTCGACGGAGCCTCAAA	TAATGGTGAGCGCGGAGAAA	Goren et al., 2011
Solyc07g042520	*SUS3*	TCACCACTACAATGGAAAGTCAA	TCTCTAGAACACGCTCTGCG	Goren et al., 2011
Solyc09g098590	*SUS4*	TCCTTGTTGTCACCCGACTG	CCTGCCACGTCCTCAGTAAA	Goren et al., 2011
Solyc11g017010	*SUT1*	GTACACTCATGGGCCCGAC	GACACCAACATCTGTGGTACA	Shen, et al. 2019
Solyc05g007190	*SUT2*	CTCTTCCTCCACCGCCTGTA	GGTTGTACCACAAGGCCAGT	Shen, et al. 2019
Solyc04g076960	*SUT4*	GGGATCCCACTAGCTATAACG	ACAAAGGCTGGTGAATTGCC	Shen, et al. 2019
XM_004237674.4	*SWEET1*	TGCCTTCTTTCTGCATGGTATG	AAACAAGGGCTACAGCAGCA	Shen, et al. 2019
Solyc03g097600	*SWEET10b*	CGTGGACAAGTTGTTGGATGG	TTGGAGCCGCGATGTTAATG	Shen, et al. 2019
Solyc03g097870	*SWEET11a*	CTACGCACCAAAGAAAGCCAG	TGACTTGTCTCACAATGCCT	Shen, et al. 2019
Solyc03g097590	*SWEET12a*	CAAAGCCAGGGTCCATACTATAA	CCACACTCTTTGTTTTGATGACT	Shen, et al. 2019

Table S2 Effects of additional far red on the parameters of the Gompertz fruit growth curve of tomato (Solanum lycopersicum) in the greenhouse experiment.

	Unit	RB+FR	RB	P-value
k		0.10	0.10	n.s.
t_m	days	22.76	24.91	P<0.01
W_{max}	g fruit^{-1}	5.59	4.01	P<0.01

P-value was calculated by ANOVA (n=4, α=0.05).

Table S3 Effect of earlier flowering on simulation of dry mass partitioning to fruits in tomato (Solanum lycopersicum) for a period of 90 days after transplanting.

Pruning	Simulated dry mass partitioning		
	Reference	1 day early	2 days early
5 fruits per truss	32.9%	33.4%	33.9%
No pruning	39.5%	39.6%	40.2%

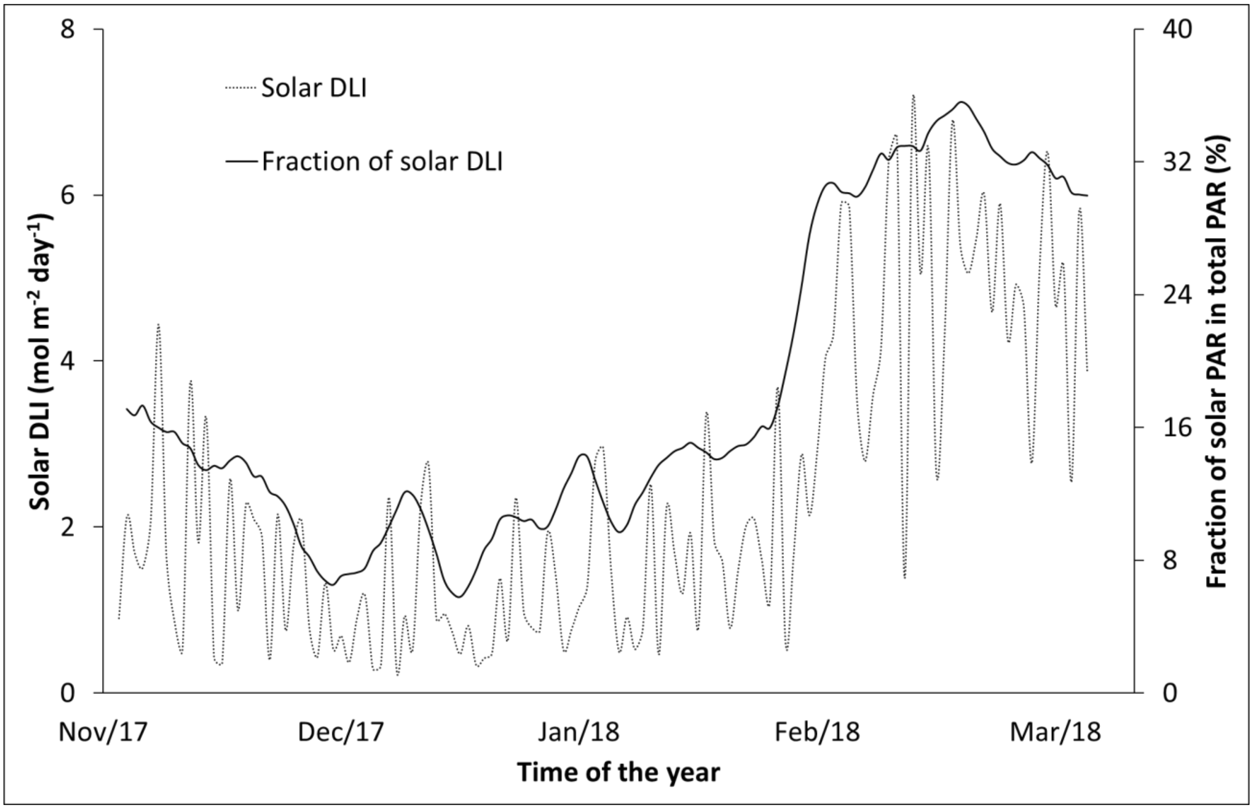

Fig.S1 Time course of solar daily light integral (DLI) of photosynthetically active radiation (PAR) and its fraction of total DLI of photosynthetically active radiation measured at the canopy level during the experiment. The dashed line represents solar DLI reaching the canopy, calculated with solar radiation measured at the roof of greenhouse and the transmissivity and shading coefficient of the greenhouse. The solid line represents a 5-day average of fraction of solar DLI in the total DLI of PAR at the canopy level.

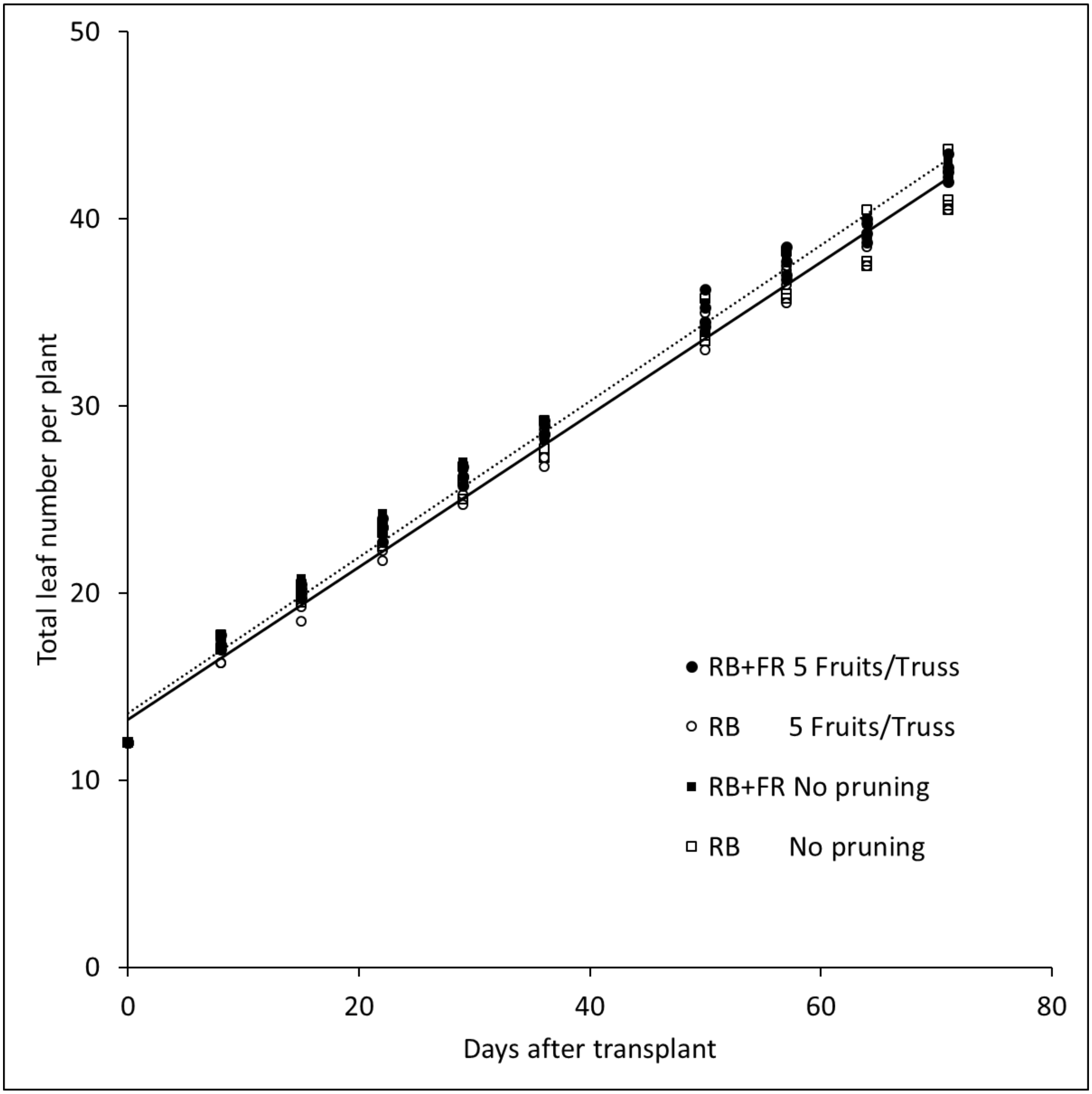

Fig.S2 Leaf number of plants of tomato (Solanum lycopersicum) plants grown with or without additional far red with 5 fruits/truss or without fruit pruning. Symbols represent actual measured leaf number and lines (dashed for RB+FR, solid for RB) were fitted with linear regression.

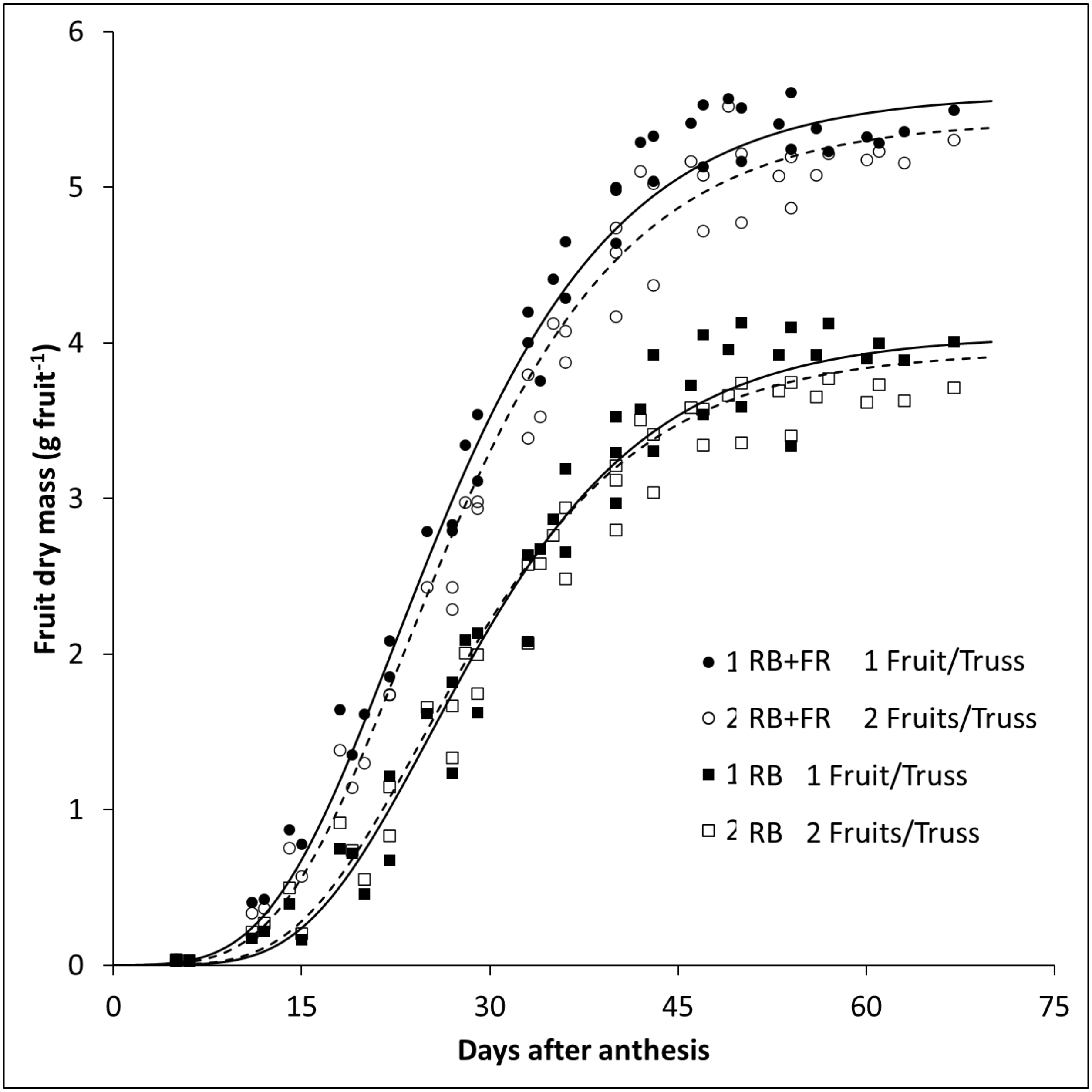

Fig.S3 Fruit growth of tomato (Solanum lycopersicum) plants grown with or without additional far red with one or two fruits per truss. Symbols represent actual measured fruit growth data and lines represent Gompertz growth curve fitted for the measured data points.

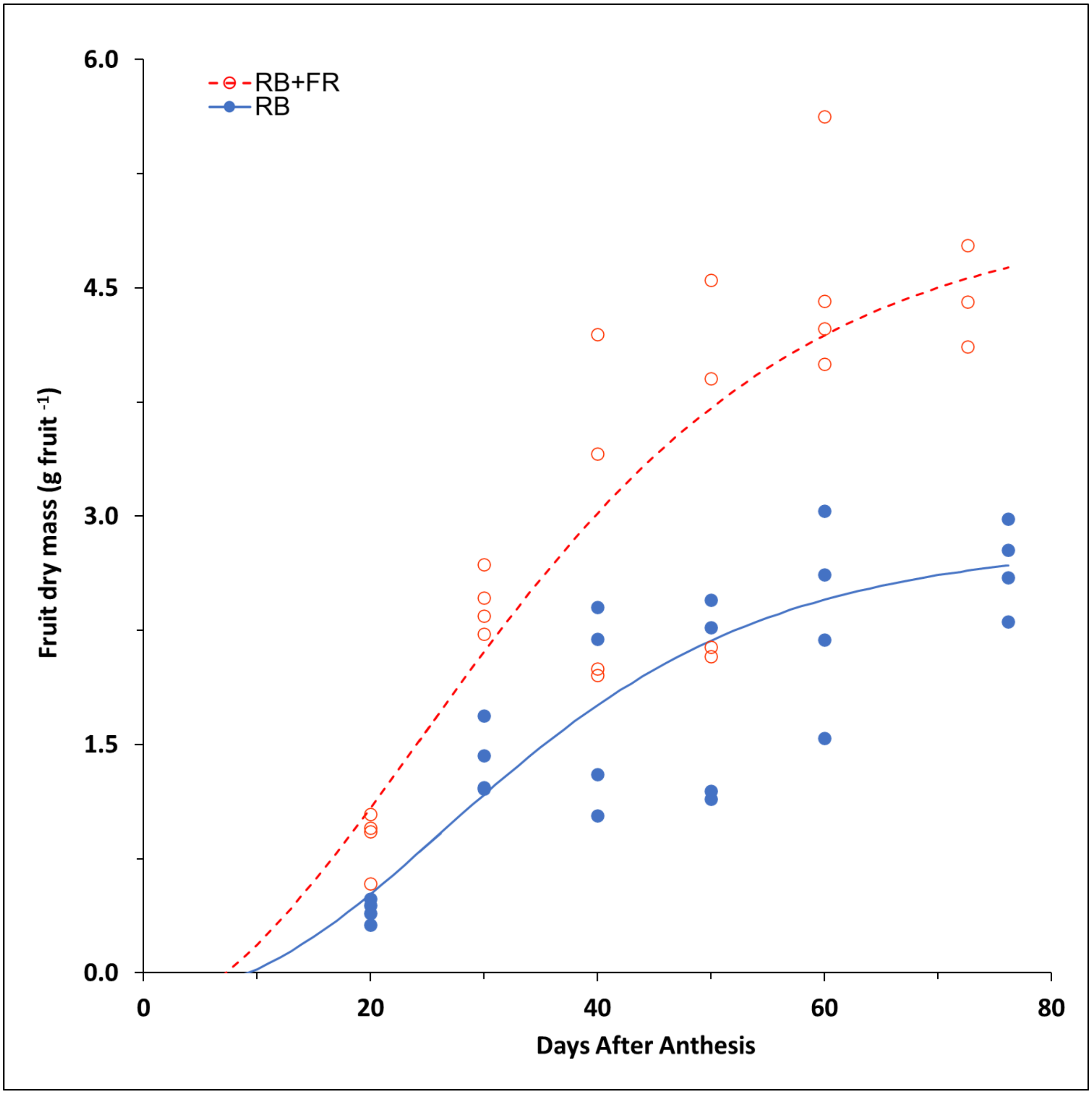

Fig.S4 Effects of adding far red (FR) to red + blue (RB) light on potential fruit growth in tomato (Solanum lycopersicum) in the climate chamber experiment. Curves represent Gompertz function fitted for RB+FR (dashed lines) and RB (solid lines) light conditions.

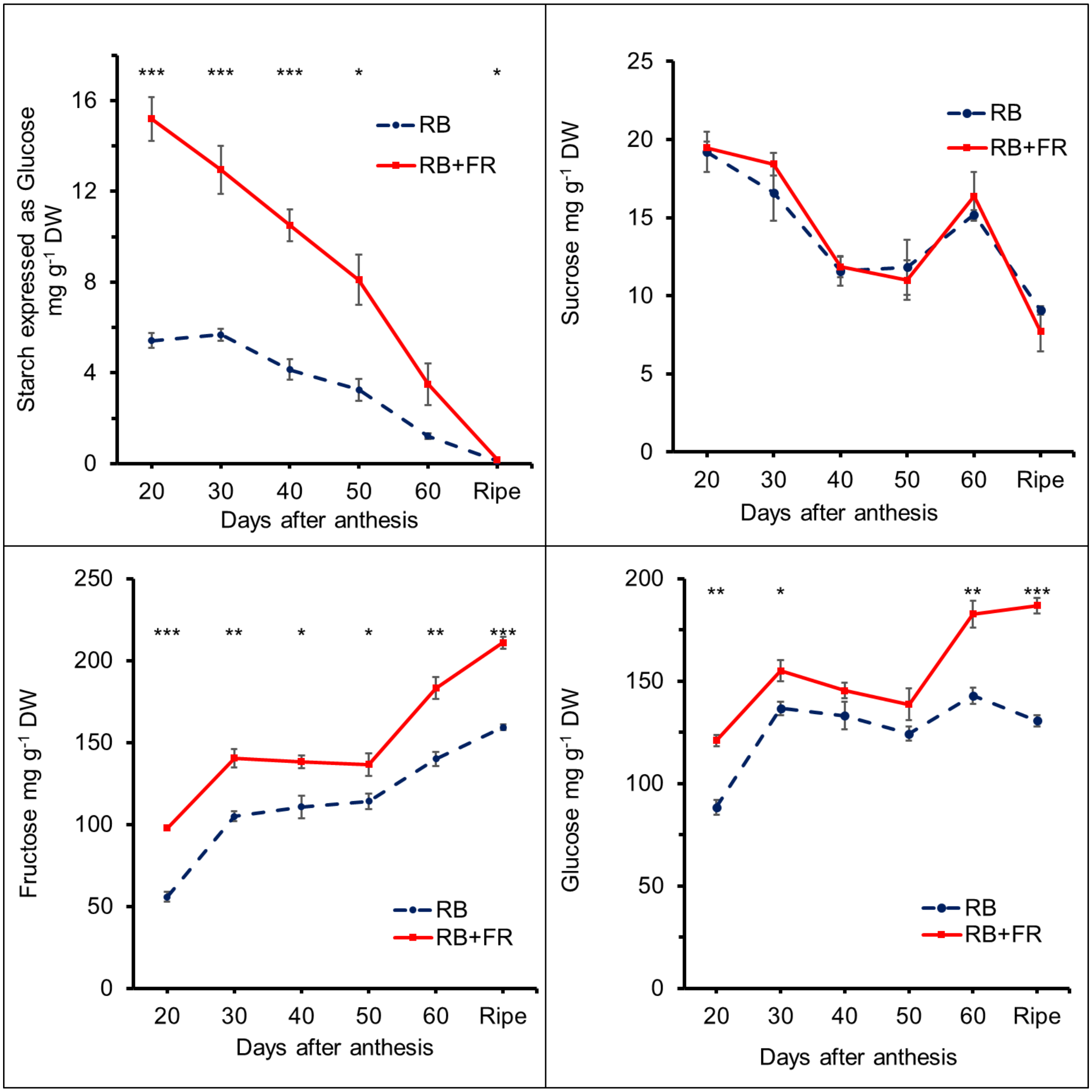

*Fig.S5 Effects of adding far red (FR) to red + blue (RB) light on concentration (mg g^{-1} fruit dry weight) of starch, sucrose, fructose and glucose in tomato(Solanum lycopersicum) fruits measured every 10 days after anthesis(DAA) until fully ripe. Starch concentration is expresses as equivalent glucose concentration. Error bar represents standard error of means (n=4). Asterisks denote statistically significant effects of FR as tested with Student's t-test (n=4, * P<0.05, ** P<0.01, *** P<0.001).*

References

Bianchetti RE, Lira BS, Monteiro SS, Demarco D, Purgatto E, Rothan C, Rossi M, Freschi L. **2018**. Fruit-localized phytochromes regulate plastid biogenesis, starch synthesis, and carotenoid metabolism in tomato. *Journal of Experimental Botany* **69**: 3573–3586.

Expósito-Rodríguez M, Borges AA, Borges-Pérez A, Pérez JA. **2008**. Selection of internal control genes for quantitative real-time RT-PCR studies during tomato development process. *BMC Plant Biology* **8**: 1–12.

Fridman E, Zamir D. **2003**. Functional divergence of a syntenic invertase gene family in tomato, potato, and Arabidopsis. *Plant Physiology* **131**: 603–609.

Goren S, Huber SC, Granot D. **2011**. Comparison of a novel tomato sucrose synthase, *SlSUS4*, with previously described SlSUS isoforms reveals distinct sequence features and differential expression patterns in association with stem maturation. *Planta* **233**: 1011–1023.

Liu YH, Offler CE, Ruan YL. **2016**. Cell wall invertase promotes fruit set under heat stress by suppressing ROS-independent cell death. *Plant Physiology* **172**: 163–180.

Reuscher S, Akiyama M, Yasuda T, Makino H, Aoki K, Shibata D, Shiratake K. **2014**. The sugar transporter inventory of tomato: genome-wide identification and expression analysis. *Plant and Cell Physiology* **55**: 1123–1141.

Shen S, Ma S, Liu Y, Liao S, Li J, Wu L, Kartika D, Mock H-P, Ruan Y-L. **2019**. Cell wall invertase and sugar transporters are differentially activated in yomato styles and ovaries during pollination and fertilization. *Frontiers in Plant Science* **10**: 506.

Zhang YL, Zhang AH, Jiang J. **2013**. Gene expression patterns of invertase gene families and modulation of the inhibitor gene in tomato sucrose metabolism. *Genetics and Molecular Research* **12**: 3412–3420.
